# Quantum contextuality, causality and freedom of choice

**DOI:** 10.1098/rsta.2023.0009

**Published:** 2024-03-18

**Authors:** Samson Abramsky, Adán Cabello, Ehtibar N. Dzhafarov, Paweł Kurzyński

**Affiliations:** ^1^ Department of Computer Science, University College London, London, UK; ^2^ Departamento de Física Aplicada II, Universidad de Sevilla, Sevilla E-41012, Spain; ^3^ Instituto Carlos I de Física Teórica y Computacional, Universidad de Sevilla, Sevilla E-41012, Spain; ^4^ Department of Psychological Sciences, Purdue University, West Lafayette, IN, USA; ^5^ Institute of Spintronics and Quantum Information, Faculty of Physics, Adam Mickiewicz University, Uniwersytetu Poznańskiego 2, Poznań 61-614, Poland; ^6^ Centre for Quantum Technologies, National University of Singapore, 3 Science Drive 2, Singapore 117543, Singapore

**Keywords:** contextuality, quantum mechanics, causality, freedom of choice

## 1. Introduction

The theme of this issue is ‘Quantum contextuality, causality and freedom of choice.’ Contextuality, including non-locality as its special case, has long since become one of the central topics in foundations of quantum mechanics, also making inroads in computer science, psychology and linguistics. Contextuality (or lack thereof, non-contextuality) is a property of a system of measurements, broadly understood. The measurements have generally random outcomes, and, as a preliminary intuition, a system of measurements is contextual if one cannot find a joint distribution for their outcomes subject to certain constraints. A precise definition depends on how a system of measurements is represented, which can be done in several different ways.

The second notion in the theme, causality, considers measurements as events with explicit causal structure. This structure includes the dependence of measurement events on their causal past, as well as the dependence of measuring a property on other properties being measured in the same experimental run. These dependences mean that the No-Signalling or No-Disturbance principle usually adopted in applications of the notion of contextuality in physics must be modified, and the notion of contextuality itself extended correspondingly.

The third notion of the theme, freedom of choice, may look like a philosophical concept outside the purview of rigorous science. However, freedom of choice and its possible violations play an important and surprisingly tangible role in the treatments of contextuality and causality. The intuitive meaning of the freedom of choice is that one’s choice of experimental settings to study a system of measurements and the properties of this system to be measured are independent of each other.

**Figure 1.**
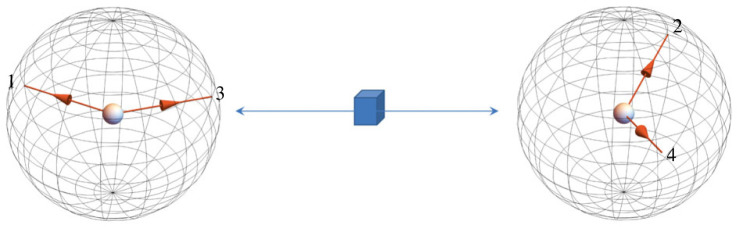
A schematic representation of the logic of the CHSH experiment (after Clauser *et al.* [1]). Two spin-$\frac{1}{2}$ particles are generated by a single source and move away from each other. The experiment involves two dichotomous measurements (with outcomes usually denoted {0,1} or {−1,1}): the left particle’s spin is measured by Alice along one of two axes, 1 or 3, and the right particle’s spin is measured by Bob along the axis 2 or 4. Thus the possible pairs of the axes used in one experimental run are {1,2}, {2,3}, {3,4}, {4,1}.

**Figure 2.**
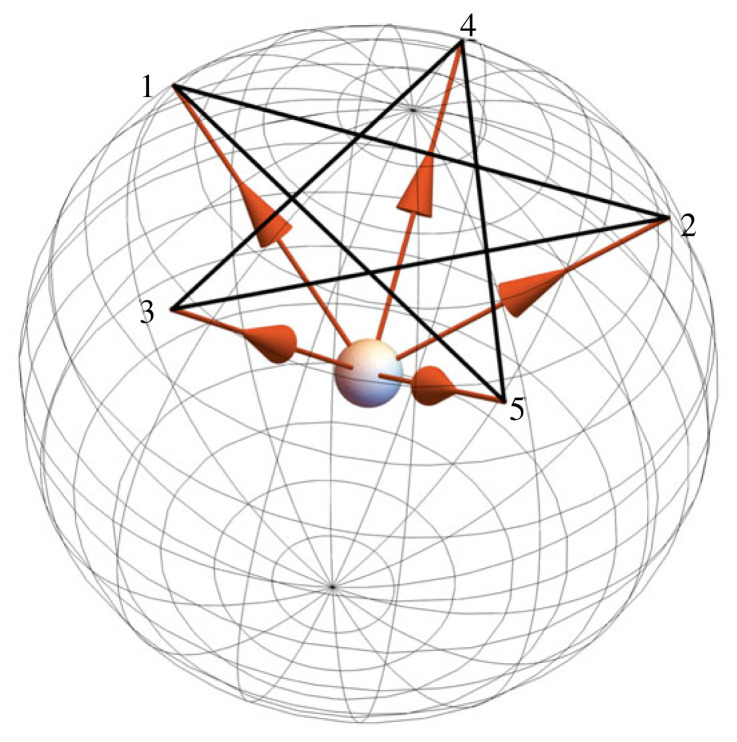
A schematic representation of the logic of the KCBS experiment (after Klyachko *et al.* [2]). The experiment involves two spin measurements made on a spin-1 particle along the pair of axes pointing at the vertices of an edge of the pentagram shown by the dark lines: the possible pairs are therefore {1,2}, {2,3}, {3,4}, {4,5}, {5,1}. Measurement outcomes are treated as dichotomous by identically labelling two of the three possible values (e.g. −1↦1, 1↦1 and 0 mapping into 0 or −1).

The papers included in this issue deal with these three theme notions in different ways, using different terminology. This terminological multiplicity is often lamented by readers of contextuality literature. For instance, non-signalling is also known under a variety of other names: non-disturbance, parameter independence, simple locality, local causality, marginal selectivity, consistent connectedness, etc. The situation is further complicated by the fact that the authors preferring a particular term usually mention conceptual reasons or semantic nuances that make other terms wanting. To partially remedy this situation, we thought it might be useful to present different theoretical accounts of the same two well-known experimental paradigms that have played an important role in contextuality research. Their descriptions are given in the captions for figures 1 and 2.

### (a) CHSH and KCBS in the sheaf-theoretic approach

A basic tenet of the sheaf-theoretic approach is to describe contextuality in a theory-neutral fashion, without presupposing Quantum Mechanics or any other theory. One can then consider which contextual systems can be realized according to Quantum Mechanics, or can arise in some other way.

There are two levels of description: the *scenario* and the *empirical model*. The scenario gives the ‘shape’ of the system, or in logical terms the *type*. It is specified as S=(X,O,C), where

— X is a set of *measurements*.
— $O={\left\{O_{x}\right\}}_{x\in X}$ is the set of possible *outcomes* for each measurement.
— C is a family of subsets of X whose union is X, which represent the *contexts* or compatible sets of measurements—those which can be performed together.

In the case of the CHSH system under consideration, we have

— X={1,2,3,4},
— $O_{x}=\{+1,-1\}$ for all x∈X,
— C={{1,2},{2,3},{3,4},{4,1}}.

Thus there is a very direct correspondence with the items given in the concrete description of the experiment.

The next level of description is that of an *empirical model* for a given scenario, which describes the actual behaviour of a specific system. This itself can be seen as having two levels.

Firstly, in a given context C∈C, there is the representation of the possible joint outcomes of performing the measurements in that context. Such a joint outcome is represented by a function which assigns, to each x∈C, an outcome in $O_{x}$.^1^

In the case of the CHSH example, we can list the set of such functions, e.g. for the context {1,2}—there are four of them

$$\begin{array}{rl} & \;\{1\mapsto +1,2\mapsto +1\},\;\{1\mapsto +1,2\mapsto -1\},\;\{1\mapsto -1,2\mapsto +1\}\;\mathrm{and}\;\{1\mapsto -1,2\mapsto -1\}. \end{array}$$

We can similarly list the joint outcomes for the other contexts. We write $E(C):=\prod_{x\in X}O_{x}$ for the set of such functions for a context C.

An important operation is that of *restriction* from a larger context to a smaller one. For example, if we have the joint outcome {2↦+1,3↦−1} for the context {2,3}, then we can restrict this to {2↦+1} for the sub-context {2}. This is important because it allows us to compare the behaviour of the system in different contexts at the points where they overlap. For example, we can also restrict the joint outcome {1↦+1,2↦+1} to the sub-context {2}, and we see that this also yields the same outcome {2↦+1}. We shall use the notation $s{|}_{C}$ where $s\in E(C^{\prime })$ and $C\subseteq C^{\prime }$, for the restriction of s to C.

The E construction only allows us to speak of *deterministic outcomes* of measurements. To represent more general behaviours, we consider *distributions* over events. Given a context C, we consider DE(C), the set of distributions over joint outcomes for the measurements in the context.^2^ In the CHSH example, let us consider the context C={1,2}. We can exhibit a distribution in DE(C) as follows:


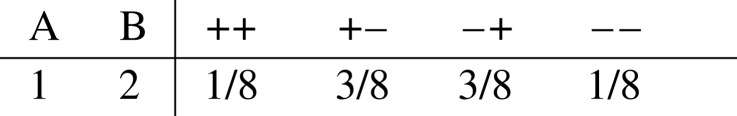

Here, the context is shown on the left-hand side of the table. By the usual convention, measurement 1 is associated with Alice, and 2 with Bob. The outcomes are used to label the columns; for brevity we write +− instead of +1−1, etc. Thus the second entry in the main part of the table says that the joint outcome {1↦+1,2↦−1} is assigned probability 3/8 by the distribution. Note that the probabilities sum to 1, as they should for a normalized distribution.

We can now say what an *empirical model* is. It specifies, for each context C∈C, a distribution $e_{C}\in DE(C)$. We can display an empirical model for the CHSH experiment as follows:


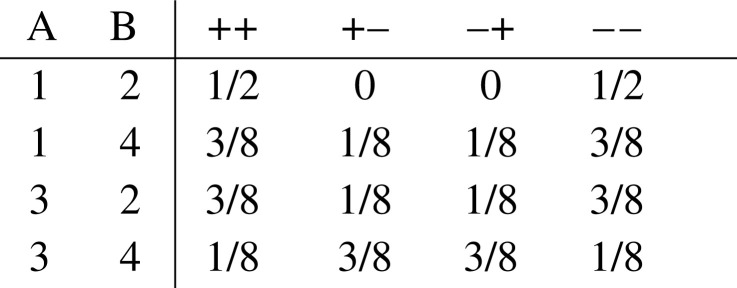

Each row of the table gives a context, and the distribution on joint outcomes for the context specified by the model. The reason for the strange-looking arrangement of the contexts will be explained in the final paragraph.

This particular empirical model can be realized in quantum mechanics, using a suitable entangled state, and appropriate choices of angles for the spin measurements. Moreover, such realizations have been experimentally verified.

To ensure compatibility with relativity, or for other reasons, a standard requirement made of empirical models is that they satisfy a condition variously known in different settings as non-signalling, non-disturbance, etc. We prefer to view it as a *local consistency* condition. Each context provides a partial view or window on the system, and local consistency is the requirement that these views agree on their overlap. This is expressed in terms of the restriction maps we have already mentioned. For the deterministic case of single assignments of joint outcomes, where we have an assignment $s_{C}\in E(C)$ for each context C∈C, the local consistency condition is expressed as follows: for each pair of contexts C and $C^{\prime }$, $s_{C}{|}_{C\cap C^{\prime }}=s_{C^{\prime }}{|}_{C\cap C^{\prime }}$. Thus two functions defined on different sets of variables are locally consistent if they assign the same outcomes to each of the variables in the intersection of their domains.

The same idea can be carried over to probabilistic models. Given contexts $C\subseteq C^{\prime }$ and a distribution $e_{C^{\prime }}\in DE(C^{\prime })$, we can define the restriction $e_{C^{\prime }}{|}_{C}\in DE(C)$. This can be understood as the marginal of the distribution $e_{C^{\prime }}$, where we project onto the smaller context C.^3^ The local consistency condition for empirical models is then expressed in exactly the same fashion as in the deterministic case: an empirical model $\{e_{C}\in DE{\left(C)\right\}}_{C\in C}$ is locally consistent if, for all $C,C^{\prime }\in C$, $e_{C}{|}_{C\cap C^{\prime }}=e_{C^{\prime }}{|}_{C\cap C^{\prime }}$.

We can check that the CHSH empirical model displayed above satisfies this property.

We now come to how contextuality, or in this case, non-locality, is defined in our approach. We have just defined the local consistency property: pieces of the model fit together locally. From a classical point of view, these views or snapshots afforded to us by contexts are views *of* a single underlying reality, which is independent of our observations of it. How can this ‘single underlying reality’ be expressed? By a single distribution d∈DE(X) on *all* the variables, without regard to the compatibility of the corresponding measurements. This distribution should allow us to recover all the observable phenomena from the empirical model ${\left\{e_{C}\right\}}_{C\in C}$. This is expressed by saying that for all contexts C, the marginal $d{|}_{C}=e_{C}$. We can think of the global distribution as ‘having all the answers’. Whichever question (context) we ask it, it provides answers indistinguishable from those we would observe if we performed the measurements in that context. The existence of such a global distribution is equivalent to the more traditional formulation in terms of hidden variables.

Thus we say that an empirical model is *non-contextual* or *globally consistent* exactly if there exists such a global distribution. If no such distribution exists, then we say that the model is contextual. Thus contextuality arises exactly when we have a system which is *locally consistent, but globally inconsistent*.^4^

The CHSH system is famous because it gives an example of contextuality (more specifically, non-locality). It can be shown, using the CHSH inequality, that no global distribution satisfying the above property can exist, so the system is globally inconsistent.

A very similar account can be given for the KCBS system. In this case, the scenario is

— X={1,2,3,4,5},
— $O_{x}=\{+1,-1\}$ for all x∈X,
— C={{1,2},{2,3},{3,4},{4,5},{5,1}}.

To make the contextuality argument for this system, it turns out that only one outcome need be considered, say +−. There is a quantum realization of an empirical model, using a qutrit state and suitably chosen observables, which witnesses contextuality for this system. This empirical model ${\left\{e_{C}\right\}}_{C\in C}$ assigns the following probabilities to the +− outcome for each context:


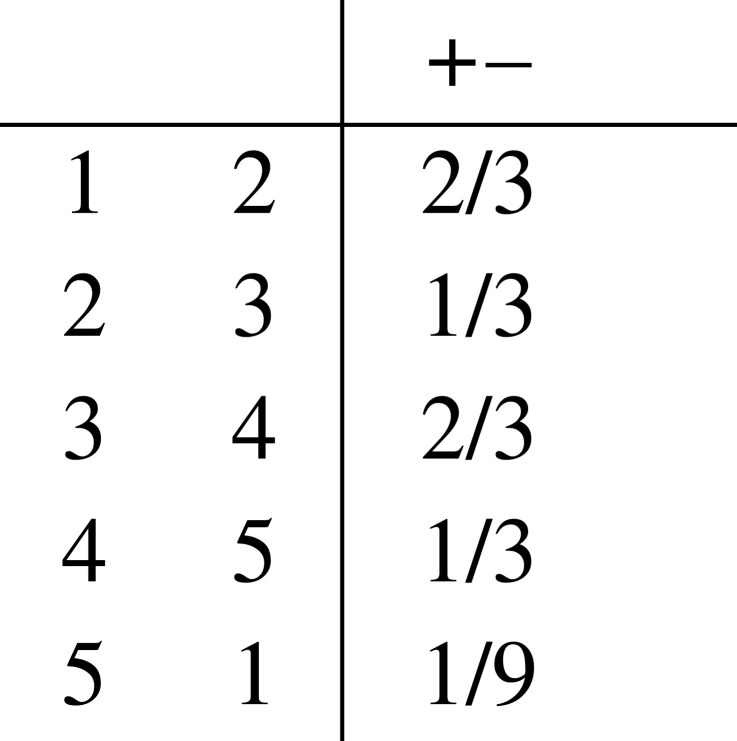

These assignments violate the KCBS inequality, showing that there is no global distribution for this empirical model, so the system is globally inconsistent/contextual.

One should note an important difference between the CHSH and KCBS systems, which is visible at the level of the scenarios. At first sight, they look similar, one being a 4-cycle and the other a 5-cycle. However, the CHSH system can be decomposed into two sets of measurements, {1,3} and {2,4}, such that {1,3} can be assigned to Alice, and {2,4} to Bob. The contexts are exactly those that arise by choosing one Alice measurement and one Bob measurement. This fits with the physical picture where Alice and Bob are spatially separated, and each performs one measurement. No such decomposition is possible for the KCBS system. The corresponding experiment is performed on a single system at one location.

### (b) CHSH and KCBS in the graph-theoretic approach

In physics, a *measurement scenario* is characterized by a set of observables, the outcomes of each observable, and the relations of compatibility between the observables. Two observables x and y are *compatible* (or *jointly measurable* or *co-measurable*) if they have a common refinement z. That is, if there exists an observable z such that x and y can be jointly measured by measuring z. A *context* is a set of compatible observables. For a given scenario, a *correlation* (*behaviour* or *empirical model*) is a set of probability distributions, one for each context.

The graph-theoretic approach to correlations focuses on two types of scenarios:

— *Kochen-Specker* (*KS*) *scenarios*, defined as those in which all the measurements are *ideal* (i.e. minimally disturbing—they only disturb incompatible observables—and repeatable—they yield the same outcome when they are measured again on the same physical system).
— *Bell scenarios*, defined as those in which there are two or more spatially separated physical systems and all the observables are *local*, i.e. refer to only one of the physical systems.

Correlations in KS and Bell scenarios are *non-disturbing* since the marginal probabilities for the outcomes of any subset of compatible observables do not depend on the context. By appealing to classical physics, in KS and Bell scenarios, it is justified to assume that there exists a single joint probability distribution that, by marginalization, produces the probability distributions of the contexts. If this is the case, then the correlations are said to be *KS non-contextual* or *Bell local*, depending on the type of scenario.

The set of KS non-contextual (Bell local) correlations for a scenario defines a polytope called the *non-contextual* (*local*) *polytope*. Each of its facets can be expressed as a weighted sum of probabilities of some events equals a constant. An *event* is characterized by the results of the measurements of the observables of a context. Each of the facets defines a tight *KS non-contextuality* (*Bell*) *inequality* and the correlations that violate one of them are called *KS contextual* (*Bell non-local*) correlations.

**Figure 3.**
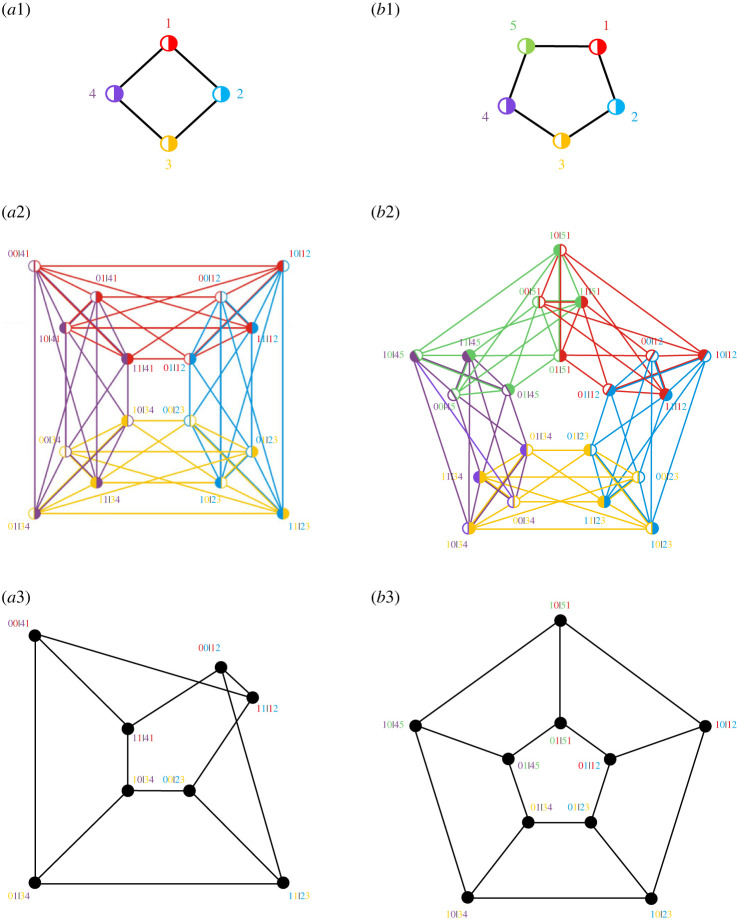
(*a*1) Compatibility graph of the CHSH scenario. Nodes represent observables and edges connect compatible observables. Nodes are divided into two parts to indicate that each observable has two outcomes. (*a*2) Exclusivity graph of the 16 events of the CHSH scenario. Here, nodes represent events. ab|xy is the event in which outcomes a and b are obtained when (compatible) observables x and y are jointly measured. Here, edges connect mutually exclusive events. The colours of the edges indicate the reason of the exclusivity. For example, a red edge indicates that the exclusivity is due to the fact that the observable represented in red (observable 1) has different outcomes in the two events. (*a*3) Exclusivity subgraph G of the eight events whose sum of probabilities S appears in the CHSH inequality. The CHSH inequality states that, for every KS non-contextual (Bell local) theory, S≤3, where 3 is the independence number of G. The maximum value of S in quantum theory is $2+\sqrt{2}\approx 3.414$, which is the Lovász theta number of G. (*b*1) Compatibility graph of the KCBS scenario. (*b*2) Exclusivity graph of the 20 events of the KCBS scenario. (*b*3) Exclusivity subgraph H of the 10 events whose sum of probabilities K appears in the KCBS inequality. The KCBS inequality states that, for every KS non-contextual theory, K≤4, where 4 is the independence number of H. The maximum value of K in quantum theory is $2\sqrt{5}\approx 4.472$, which is the Lovász theta number of H.

The *compatibility graph* of a scenario represents observables by nodes and compatibility relations by edges. A node may be divided into d parts to indicate that the corresponding observable has d outcomes. Vorob’ev’s theorem states that KS contextuality and Bell non-locality are impossible in any scenario whose compatibility graph does not contain induced cycles of size larger than three. As an illustration, figure 3*a*1,*b*1 shows the compatibility graphs of the CHSH and KCBS scenarios, respectively.

Two events are *mutually exclusive* if they contain an observable that has different outcomes in the two events. The *exclusivity graph* of a given KS or Bell scenario represents the events of that scenario by nodes and their exclusivity relationships by edges. As an illustration, figure 3*a*2,*b*2 shows the exclusivity graphs of the events of the CHSH and KCBS scenarios, respectively.

The interest in exclusivity graphs stems from the observation that the set of non-contextual (quantum) correlations for a KS or Bell scenario is a subset of the stable set polytope (theta body) of the exclusivity graph. Since any non-contextuality and Bell inequality can be expressed as a weighted sum of probabilities of a subset of events, the non-contextual (local) bound of a non-contextuality (Bell) inequality is the independence number of the vertex-weighted induced subgraph G of the exclusivity graph of these events. Similarly, the maximum quantum value is upper bounded by the Lovász theta number of G. As an illustration, figure 3*a*3,*b*3 shows the exclusivity graphs associated with the CHSH Bell inequality and the KCBS non-contextuality inequality, respectively, which are the only non-trivial tight non-contextuality inequalities in the CHSH and KCBS scenarios, respectively.

### (c) CHSH and KCBS through negative probabilities

Quantum contextuality can be explained through the Hilbert space formalism. However, there is an alternative explanation using a single distribution approach. As mentioned above, contextuality implies lack of a single probability distribution over all measurements. However, it is still possible to construct a single joint quasi-probability distribution for contextual scenarios. Such quasi-probabilities admit negative values, hence it is customary to simply call them negative probabilities. Therefore, if a system exhibits contextuality, then the corresponding joint distribution is negative. What is important, these negative probabilities are unobservable since in experiments one is only able to detect marginal distributions, which are proper non-negative probability distributions.

For example, in the CHSH scenario discussed above a joint distribution is of the form p(a,b,c,d|A,B,C,D) (p(a,b,c,d) to simplify notation), where A, B, C and D correspond to the axes 1, 2, 3 and 4 from figure 1, respectively, and a,b,c,d=±1 are specific outcomes of these measurements. Let us consider the following one:

1.1 $$p(a,b,c,d)=\frac{1}{16}\left[1+\frac{\lambda }{2}(ab+ad+cb-cd)\right]$$

that is parameterized by λ≥0. Note that

1.2 $$\sum_{a,b,c,d=\pm 1}p(a,b,c,d)=1$$

and

1.3 $$p(a,b)=\sum_{c,d=\pm 1}p(a,b,c,d)=\frac{1}{4}\left(1+\frac{\lambda }{2}ab\right).$$

The marginal probabilities mentioned above are non-negative when λ≤2. The same holds for marginal distributions p(a,d), p(c,b) and p(c,d). However, for 1≤λ≤2, certain joint probabilities become negative, for instance, $p(-1,-1,-1,-1)=\frac{1}{16}(1-\lambda )$.

A similar approach can be taken for the KCBS scenario. Consider a distribution

1.4 $$p(a,b,c,d,e)=\frac{1}{32}\left[1-\frac{\lambda }{2}(ab+bc+cd+de+ea)\right],$$

for λ≥0. This time a, b, c, d and e are outcomes of the measurements represented in figure 2 as the axes 1, 2, 3, 4 and 5, respectively. The above distribution gives rise to the observable marginal distribution

1.5 $$p(a,b)=\frac{1}{4}\left(1-\frac{\lambda }{2}ab\right),$$

and similar for p(b,c), p(c,d), p(d,e) and p(e,a). Marginal distributions are non-negative for λ≤2. However, for $\frac{2}{5}\leq \lambda \leq 2$, the joint distribution is negative.

### (d) CHSH and KCBS in the Contextuality-by-Default approach

In the theory called Contextuality-by-Default (CbD), the scenarios CHSH and KCBS can be represented, respectively, by the following *systems of double-indexed random variables*:
1.6

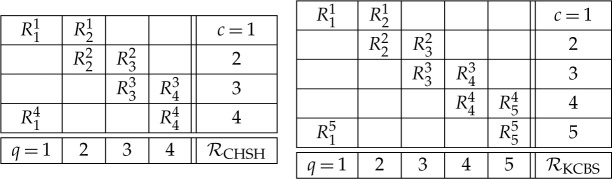

In each variable $R_{q}^{c}$, the value of q is referred to as the *content* of the variable (in this case, the measurement defined by the axis chosen), and the value of c indicates the *context* of the variable. In our systems (1.6), all the variables are binary, with values we will label −1 and 1. For any context c, the variables $\left(R_{q}^{c},R_{q^{\prime }}^{c}\right)$ form a *jointly distributed* *bunch* of variables. By contrast, variables belonging to different contexts are *stochastically unrelated*, i.e. they possess no joint distribution (e.g. there is no joint probability of $R_{1}^{1}=1$ and $R_{3}^{2}=1$). In particular, this holds for any distinct variables with the same content, $\left\{R_{q}^{c},R_{q}^{c^{\prime }}\right\}$. In CbD, the set of all variables sharing a content q is said to form a *connection* (the intuition being that they connect stochastically unrelated ‘islands’ of different bunches).

CbD uses several other terms derived from the term *connection*. Thus, if the random variables within each connection are identically distributed, the system is called *consistently connected*.^5^ Another use of connectedness relates to *couplings* of systems of variables. A coupling of a system in (1.6) is a system of identically double-indexed random variables $S_{q}^{c}$ such that its bunches have the same distribution as the corresponding bunches in the system; unlike in the latter, however, *all* variables in the coupling, even across contexts, are jointly distributed. A coupling is called (*multi*)*maximally connected* if, in each of its connections, any two random variables coincide with the maximal possible probability.^6^ A system is considered *non-contextual* if it has such a (multi)maximally connected coupling. Otherwise the system is *contextual*.

If the system is consistently connected (in CbD, it does not have to be), the maximal probability in the definition of non-contextuality is one. In this case, we can simply say that the systems in (1.6) are non-contextual if they have couplings of the following structure:
1.7

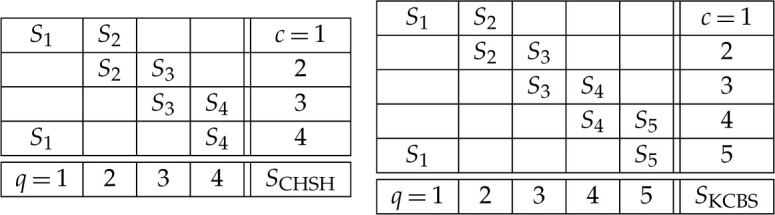


The existence or non-existence of a multimaximal coupling for any system with the finite number of variables is effectively established by means of linear programing. In the simplest cases, such as the CHSH and KCBS experiments, one can also derive a set of inequalities (referred to as *Bell-type inequalities*) that are satisfied if and only if the couplings in question exist. The Bell-type inequalities for these systems are as follows:

1.8 $$\max_{\prod \iota_{c}=-1}\sum_{c=1}^{N}\iota_{c}\left\langle R_{q=c}^{c}R_{q=c⊕1}^{c}\right\rangle \leq n-2+\sum_{q=1}^{N}\left|\left\langle R_{q}^{c=q}\right\rangle -\left\langle R_{q}^{c=q⊖1}\right\rangle \right|,$$

where ⟨⋯⟩ denotes expected value, ⊕1 and ⊖1 are clockwise and counterclockwise shifts on the dial with 1,2,…,N (with N=4 in CHSH and N=5 in KCBS), and $\iota_{c}=\pm 1$.

The systems in (1.6) are not the only way to represent the CHSH and KCBS experiments. CbD allows for other ways of defining contents and contexts, some of which, however, are less interesting for contextuality analysis. There is, however, a representation that is *contextually equivalent* to (1.6). We show it here for the CHSH experiment only
1.9

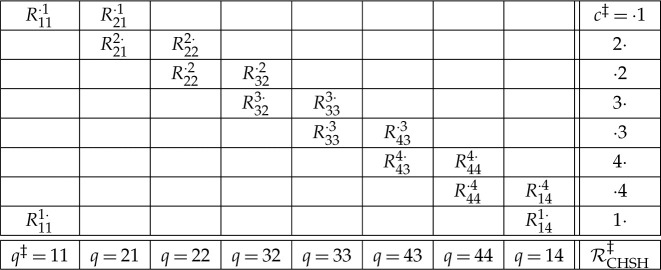

Here, the new contents $q^{‡}$ and new contexts $c^{‡}$ are defined by relating them to the contents q and contexts c in $R_{\mathrm{CHSH}}$. Namely, $q^{‡}=ij$ corresponds to the content q=i and context c=j; the bunch in context $c^{‡}=\cdot j$ is a distributional copy of the bunch in the context c=j; and the two variables $R_{ij}^{i\cdot }$ and $R_{ij^{\prime }}^{i\cdot }$ in a context $c^{‡}=i\cdot$ are distributional copies of, respectively, $R_{i}^{j}$ and $R_{i}^{j^{\prime }}$ in $R_{\mathrm{CHSH}}$. In the latter type of context, $c^{‡}=i\cdot$, the joint distribution is created by making the two variables coincide with the maximal possible probability. The system $R_{\mathrm{CHSH}}^{‡}$ is called the *consistification* of the system $R_{\mathrm{CHSH}}$, because $R_{\mathrm{CHSH}}^{‡}$ is (strongly) consistently connected even if $R_{\mathrm{CHSH}}$ is not, although they are otherwise interchangeable in all considerations regarding contextuality.

## Data Availability

This article has no additional data.

## Footnotes

1 Technically, since the set of outcomes in general depends on the measurement, the set of such functions forms a *dependent product*: $\prod_{x\in X}O_{x}$.

2 In more sophisticated language, E is the sheaf of local sections, while D is the endofunctor part of the distribution monad. We are composing them functorially to form DE.

3 More precisely, it is the pushforward of the distribution $e_{C^{\prime }}$ along the restriction map $E(C^{\prime })\to E(C)$.

4 In the CbD framework also presented in this introduction, contextuality is extended beyond the locally consistent case.

5 A system is called *strongly consistently connected* if whenever two bunches have a set of contents in common, the corresponding random variables in the two bunches have identical joint distributions. In our examples, no two bunches have more than one content in common, which means that consistent connectedness here is always strong.

6 Our examples contain only two variables in each connection, making the suffix *multi* unnecessary.
